# Nitric Oxide Synthase Is Involved in Follicular Development via the PI3K/AKT/FoxO3a Pathway in Neonatal and Immature Rats

**DOI:** 10.3390/ani10020248

**Published:** 2020-02-05

**Authors:** Junrong Li, Wei Zhang, Shanli Zhu, Fangxiong Shi

**Affiliations:** 1 College of Agriculture and Bio-Engineering, Jinhua Polytechnic, Jinhua 321017, China; 20191040@jhc.edu.cn; 2 College of Animal Science and Technology, Nanjing Agricultural University, Nanjing 210095, China; yuntaishan2003@aliyun.com (W.Z.); fxshi@njau.edu.cn (F.S.)

**Keywords:** nitric oxide synthase, PTEN, AKT, FoxO3a, follicular development

## Abstract

It is assumed that nitric oxide synthase and nitric oxide are involved in the regulation of female reproduction. This study aimed to assess the roles of nitric oxide synthase (NOS) in follicular development. The endothelial NOS (eNOS) inhibitor L-NAME, inducible NOS (iNOS) inhibitor S-Methylisothiourea (SMT) and NOS substrate L-arginine (L-Arg) were used in the NOS inhibition models in vivo. Neonatal female rats were treated with phosphate buffer saline (PBS, control), L-NAME (L-NG-Nitroarginine Methyl Ester, 40 mg/kg), SMT (S-Methylisothiourea, 10 mg/kg), L-NAME + SMT, or L-Arg (L-arginine, 50 mg/kg) via subcutaneous (SC) injection on a daily basis for 19 consecutive days, with the samples being collected on specific postnatal days (PD5, PD10, and PD19). The results indicated that the number of antral follicles, the activity of total-NOS, iNOS, neuronal NOS (nNOS), and eNOS, and the content of NO in the ovary were significantly (*p* < 0.05) increased in the L-Arg group at PD19, while those in L + S group were significantly (*p* < 0.05) decreased. Meanwhile, the ovarian expression in the L-Arg group in terms of p-AKT, p-FoxO3a, and LC3-II on PD19 were significantly (*p* < 0.05) upregulated, while the expressions of PTEN and cleaved Caspase-3 were (*p* < 0.05) downregulated as a result of NOS/NO generation, respectively. Therefore, the results suggest that NOS is possibly involved in the maturation of follicular development to puberty via the PI3K/AKT/FoxO3a pathway, through follicular autophagia and apoptosis mechanisms.

## 1. Introduction

Nitric oxide (NO) is a key gaseous signaling molecule that functions as a biological mediator and is produced by mammalian cells [1,2,3]. NO can be generated by the enzyme nitric oxide synthase (NOS) with three different isoforms, including neuronal NOS (nNOS), inducible NOS (iNOS), and endothelial NOS (eNOS), formed from L-arginine (L-Arg) in the presence of nicotinamide-adenine-dinucleotide phosphate (NADPH) as a substrate and co-substrate, respectively [4]. Calcium/calmodulin dependent nNOS and eNOS temporarily produce a small amount of NO, whereas the synthesis of NO over a long period is generated by iNOS, which is regulated by several cytokines and hormones [5]. NO and NOS are possibly involved in the regulation of female rat reproduction [6,7], such as in the follicular development in rats [8], and may also have a role in the reproduction of sheep [9], oocyte competence in pigs and cattle [10], luteal regression in sheep [5,11], ovarian steroidogenesis in rats [12], vitellogenesis in fish [13], and advanced onset of puberty in rats [14]. Females with polycystic ovary syndrome (PCOS) have a lowered level of NO due to reduced iNOS/eNOS expression and arginine bioavailability [15]. The estrus cycle is prolonged in a female mouse model after the knockout of eNOS genes [16,17], and a role for eNOS has been suggested in the selection of nonovulatory dominant follicles in bovines [18] and in the ovulatory process in ewes [11]. NO is produced by iNOS only in immature follicles, which has a cytostatic factor in the earlier stages of rat follicular development [19]. Moreover, the ovulation rate and implantation loss are increased by disruption of eNOS and iNOS [16]. Knocking out the three isoenzymes of NOS in mice leads to a significant decline of the fertility rate, as well as the occurrence of various cardiovascular diseases [20].

The PI3K/AKT pathway is a key regulator of cell proliferation, growth, and survival [21,22]. The FoxO transcription factors, also called the forkhead transcription factors, are a subfamily including FOXO3A (FKHRL1), FOXO1A (FKHR), FOXO6, and FOXO4 (AFX), with pivotal roles in the PTEN/PI3K/AKT pathway [23]. FoxO3a has been proposed to be the key transcription factor in the PI3K/PTEN-mediated maintenance of primordial follicle reserves [24]. The PI3K pathway regulates the activation of primordial follicles via transcription factor FoxO3 [25,26,27], and it has been reported that PI3K inhibitor decreases the levels of phosphorylated FoxO3a (p-FoxO3a) while increasing the levels of nuclear FoxO3a [28]. The PI3K/AKT/FoxO3a pathway is involved in the regulation of various physiological processes via NO or NOS, guaranteeing an improved follicular microenvironment for developing oocytes and inhibiting granulosa cell apoptosis [29,30]. During the migration of endothelial cells, peroxisome proliferator-activated receptor coactivator 1α (PGC-1α) is downregulated by NO via the activation of the PI3K/protein kinase B (Akt protein) pathway and FoxO3a protein [31]. The balance of the PI3K signaling pathway is pivotal to maintain the growth and survival of the primordial follicle pool [32]; a rodent model revealed that the PI3K/AKT pathway mediated oocyte apoptosis and FoxO3a plays an important role in early follicular development [29]. Furthermore, FoxO3a was found to maintain the PTEN-PI3K-AKT-FoxO3a pathway, enabling faster follicle growth [33]. Previous researchers have found that locally produced NO and the NOS/sGC pathway might be involved in the maturation of the follicles [34,35]. However, the mechanism of the PI3K/AKT/FoxO3a pathway via NO/NOS participation in the follicular development of neonatal and immature animals has not been reported yet, and requires further investigation.

Previous studies on the mechanisms of NOS inhibitor injection either in vitro or in vivo, including L-NG-Nitroarginine Methyl Ester (L-NAME) and S-Methylisothiourea (SMT) as eNOS-selective (non-selective at high level), and iNOS-selective inhibitors, respectively [36,37,38,39], have proposed that L-NAME would significantly reduce cyclic guanosine monophosphate (cGMP) production in follicles in vitro [40], impair folliculogenesis, and delay ovarian follicular development [41]. In addition, the offspring of pregnant rats which underwent in utero exposure to L-NAME exhibit decreased neonatal weight, postnatal growth, and fertility [42]. Meanwhile, NOS substrate L-Arg is widely used to produce NO [43,44,45]. Finally, in recent decades, an increasing number of studies have concentrated on the effects of NOS on the reproductive system of female rats. So far, there has been no study to evaluate the effects of NOS on follicular development in neonatal and immature rats. Therefore, we herein adopted subcutaneous administration of the L-NAME, SMT, or L-Arg to generate a NOS inhibition model in rats, and clarify the effects of NOS on follicular development via the regulating mechanisms of the NO/cGMP and PI3K pathways.

## 2. Materials and Methods

### 2.1. Experimental Design and Ethics Statement

Ten male and ten female mature Sprague–Dawley rats were selected for the experiment and kept at a room temperature of 22 ± 1 °C and relative humidity of 30–40%. Females and males were randomly matched and mated (1:1), after which the female rats gave birth to neonatal female rats. The individuals (body weight 4–5 g) from each litter were randomly assigned to five groups (*n* = 5). The first day of birth was numbered as postnatal day 1 (PD1). The neonatal female rats from the five groups were subcutaneously injected with 50 μL phosphate buffer saline (PBS, control), a solution of L-NG-Nitroarginine Methyl Ester (L-NAME, 40 mg/kg), S-Methylisothiourea (SMT, 10 mg/kg), L-NAME plus SMT (L + S), or L-Arginine (L-Arg, 50 mg/kg) daily in the morning from PD1 for 19 consecutive days. The animals were euthanized by CO_2_ anesthesia on PD5, PD10, or PD19 (12 h after injection), and the ovaries collected under stereomicroscopy. The right-side ovary was fixed in 4% paraformaldehyde for hematoxylin-eosin staining (HE), while the left-side samples were measured for NOS activity and NO concentration before they were stored at −80 °C for Western blotting analysis (WB). The experiment procedures conformed to the guidelines for the care and use of experimental animals issued by the Animal Ethical and Welfare Committee of Jinhua Polytechnic (approval number: 20170609-01), China.

### 2.2. Histological and Morphological Examination

The samples were fixed for 24 h and then embedded in paraffin wax and sectioned serially at 4 μm. The HE tissues were stained with hematoxylin and eosin (Nanjing Jiancheng Bioengineering Institute, Nanjing, China). The follicles were counted by evaluating six slices per sample (randomly 10–15 slices interval with different number of follicles), and were then divided into unassembled follicles, primordial follicles, primary follicles, secondary follicles, and tertiary follicles (antral follicles) [46]. Each primordial follicle consisted of a layer of flat follicular cells and an immature oocyte. After the follicular cells outside the oocyte turned from a flat shape into a cuboid one, a primary follicle was formed. A secondary follicle was formed with the gradual increase of granulosa cell layers [17]. At the stage of tertiary follicles, the granulosa cells differentiated into multiple layers and formed cavities; these are also called antral follicles.

### 2.3. Measurement of NOS Activity and NO Concentration

The ovaries were weighed and homogenized in iced saline (tissue weight/lysis buffer weight 1:10 suspension), then centrifuged for 10 min (2500 r) at 4 °C. The activities of total NOS, iNOS, and cNOS (eNOS + nNOS) were measured with a commercial NOS-typed assay kit (the inter- and intra-coefficient of variation of assays were respectively 2.10% and 6.01%, detection limit: 0.2–81.9 U/mL) (Nanjing Jiancheng Bioengineering Institute, Nanjing, China). The ovarian protein homogenates (800 μg/mL) were treated with nNOS inhibitor spermidine trihydrochloride (120 μmol/mL, C_7_H_19_N_3_·3HCl) to further address the eNOS activity [34], and the optical density was measured at 530 nm by an ELISA reader (*n* = 5) (BioTek Instruments, Inc., Winooski, VT, USA) based on the release of lactate NO generated by a 5–electron oxidation of terminal guanidinium nitrogen on L-arginine [47].

### 2.4. Western Blotting

The samples stored at −80 °C were homogenized in radio-immunoprecipitation assay (RIPA) buffer with 10 mM PMSF. An equal amount of protein lysate (50 μg) was separated by 12% (w/v) sodium dodecyl sulfate polyacrylamide gel electrophoresis (SDS-PAGE, Sangon Biotech, Shanghai, China), and electro-transferred onto polyvinylidene fluoride (PVDF) membranes (Millipore, Burlington, MA, USA). The membranes were blocked with 3% BSA (BBI, Shanghai, China) for 2 h at 25 °C, and incubated with primary antibodies (diluted in PBS) of β-tubulin (1:5000, ab6046, Abcam, Cambridge, UK), PTEN (1:1000, sc-7974, Santa Cruz Biotechnology, Inc., Santa Cruz, CA, USA), P-AKT (1:500, ab207452, Abcam, Cambridge, UK), AKT (1:1000, 9272, Cell Signaling, Danvers, MA, USA), p-FoxO3a (1:1000, 9464, Cell Signaling, Danvers, MA, USA), FoxO3a (1:1000, ab47285, Abcam, Cambridge, UK), cleaved Caspase-3 (1:2000, 9661, Cell Signaling, Danvers, MA, USA), and LC3-II (1:1000, 3868, Cell Signaling, Danvers, MA, USA) for 16 h at 4 °C. The incubated membranes were washed with fresh TBST (Tris-Buffered Saline Tween) buffer and incubated with HRP (Horseradish Peroxidase)-conjugated secondary donkey anti-goat IgG antibody (1:3000, A0181, Beyotime, Haimen, Jiangsu, China), followed by goat anti-rabbit IgG antibody (1:3000, SN134, Sunshine, China) at room temperature for 2 h, respectively. The bands were washed in TBST buffer (five times) and detected with a Pierce ECL WB Substrate (Thermo Scientific, Waltham, MA, USA), and visualized by a Luminescent Image Analyzer LAS4000 (Fuji Film, Tokio, Japan). The signal intensity was plotted as the ratio of the target protein to β-tubulin.

### 2.5. Statistical Analysis

All numerical results are expressed as mean ± standard error of the mean (mean ± SEM). Data were analyzed by one-way analysis of variance (ANOVA) followed by Tukey’s multiple comparison test using SPSS version 13.0, with *p* < 0.05 being considered as significant statistical difference.

## 3. Results

### 3.1. Follicular Development

**Figure 1:**
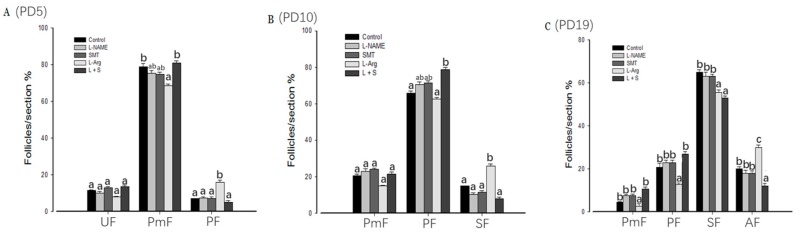
Effect of nitric oxide synthase (NOS) inhibitors and substrate on follicular development in neonatal and immature rats. Animals in the control, L-NG-Nitroarginine Methyl Ester (L-NAME), S-Methylisothiourea (SMT), L-arginine (L-Arg), or L + S (L-NAME plus SMT) groups were subcutaneously hypodermically injected with phosphate buffer saline (PBS), L-NAME (40 mg/kg), SMT (10 mg/kg), L-NG-Nitroarginine Methyl Ester (40 mg/kg) and S-Methylisothiourea (10 mg/kg), or L-arginine (50 mg/kg) for five (PD5, **A**), 10 (PD10, **B**), or 19 days (PD19, **C**) from the first day of birth (PD1). UF, unassembled follicle; PmF, primordial follicle; PF, primary follicle; SF, secondary follicle; AF, antral follicle. Bars with different letters denote significant differences (*p* < 0.05).

Compared with the control group, the percent of primordial follicles in group L-Arg (Figure 1A) was significantly (*p* < 0.05) decreased on PD5, while primary follicles were significantly (*p* < 0.05) increased. The percentage of primary follicle in the L + S group was significantly (*p* < 0.05) higher than in the control group and L-Arg on PD10, and the percentage of secondary follicles in the L-Arg group was significantly (*p* < 0.05) higher than in the control group and L + S (Figure 1B). However, the percentage of secondary follicles in the L-Arg and L + S groups was significantly (*p* < 0.05) decreased on PD19, and the percentage of antral follicles was significantly increased in the L-Arg group and significantly decreased in the L + S group, respectively (*p* < 0.05, Figure 1C).

### 3.2. Ovarian NOS Activity and NO Content

**Figure 2:**
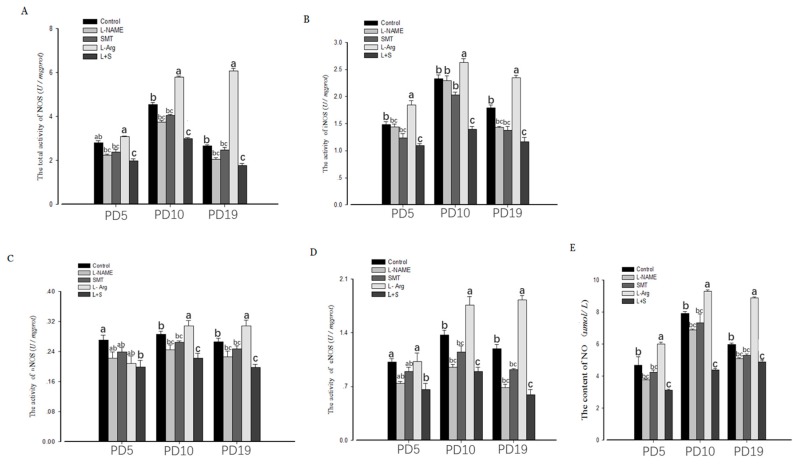
Effect of NOS inhibitors and substrate on ovarian NOS (total NOS, **A**; iNOS, **B**; nNOS, **C**; eNOS, **D**) activity and NO concentration (**E**) in neonatal and immature rats. Animals in the control, L-NAME, SMT, L-Arg or L + S (L-NAME plus SMT) groups were subcutaneously hypodermically injected with PBS, L-NG-Nitroarginine Methyl Ester (40 mg/kg), S-Methylisothiourea (10 mg/kg), or L-NG-Nitroarginine Methyl Ester (40 mg/kg) and S-Methylisothiourea (10 mg/kg), L-arginine (50 mg/kg) for five (PD5), 10 (PD10), or 19 days (PD19) from the first day of birth (PD1). Bars with different letters denote significant differences (*p* < 0.05).

The ovarian activity of total-NOS, iNOS, nNOS, and eNOS, and the content of NO, showed no significant (*p* > 0.05) differences among the control group, L-NAME and SMT (Figure 2A–E) on PD5, PD10, and PD19. Compared with the control group, the ovarian activity of total-NOS, iNOS, nNOS, and eNOS, and the content of NO, in the L + S group were significantly lower on PD5, PD10, and PD19 (*p* < 0.05, Figure 2A–E). The ovarian activity level of total-NOS, iNOS, and eNOS and the content of NO, in the L-Arg group were significantly elevated on PD10 (*p* < 0.05, Figure 2A,B,D,E), and the ovarian activity levels of total-NOS, iNOS, nNOS, and eNOS, and the content of NO, in the L-Arg group were significantly elevated on PD19 (*p* < 0.05, Figure 2A–E).

### 3.3. Ovarian Expression of PTEN, p-AKT, AKT, p-FoxO3a and FoxO3a

**Figure 3:**
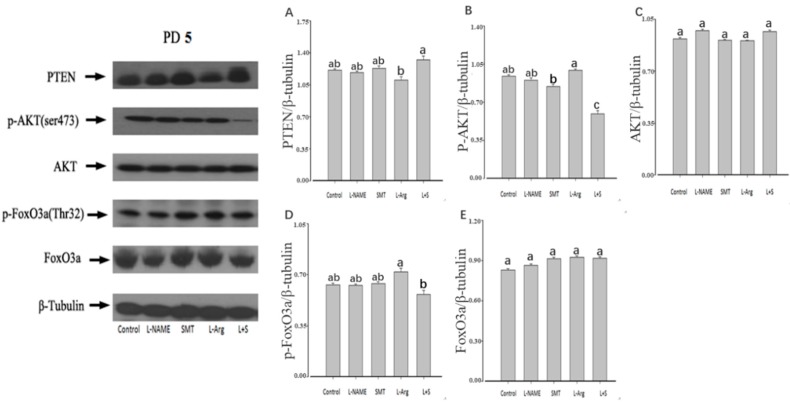
Effect of NOS inhibitors and substrate on ovarian expression of PTEN (**A**), p-AKT (**B**), AKT (**C**), p-FoxO3a (**D**), and FoxO3a (**E**) in neonatal rats. Animals in the control, L-NAME, SMT, L-Arg, or L + S (L-NAME plus SMT) groups were subcutaneously hypodermically injected with PBS, L-NG-Nitroarginine Methyl Ester (40 mg/kg), S-Methylisothiourea (10 mg/kg), L-NG-Nitroarginine Methyl Ester (40 mg/kg) and S-Methylisothiourea (10 mg/kg), or L-arginine (50 mg/kg) for five days (PD5) from the first day of birth (PD1). β-tubulin was used as an internal control, wherein the signal intensity was plotted as the ratio of target protein to β-tubulin. Bars with different letters denote significant differences (*p* < 0.05).

**Figure 4:**
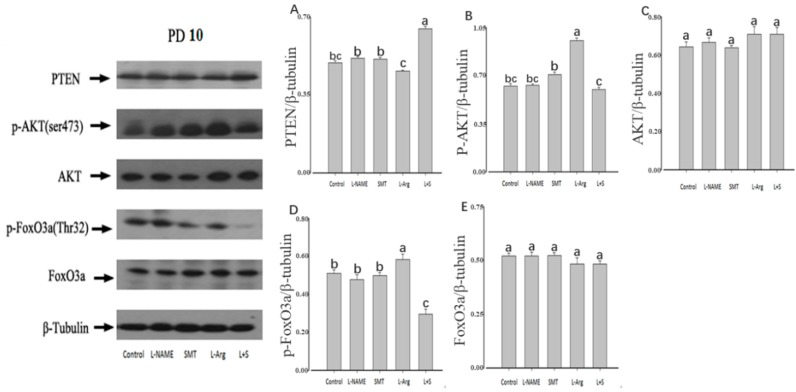
Effect of NOS inhibitors and substrate on ovarian expression of PTEN (**A**), p-AKT (**B**), AKT (**C**), p-FoxO3a (**D**), and FoxO3a (**E**) in immature rats. Animals in the control, L-NAME, SMT, L-Arg, or L + S (L-NAME plus SMT) groups were subcutaneously hypodermically injected with PBS, L-NG-Nitroarginine Methyl Ester (40 mg/kg), S-Methylisothiourea (10 mg/kg), L-NG-Nitroarginine Methyl Ester (40 mg/kg) and S-Methylisothiourea (10 mg/kg), or L-arginine (50 mg/kg) for 10 days (PD10) from the first day of birth (PD1). As an internal control, β-tubulin was used, wherein the signal intensity was plotted as the ratio of target protein to β-tubulin. Bars with different letters denote significant differences (*p* < 0.05).

**Figure 5:**
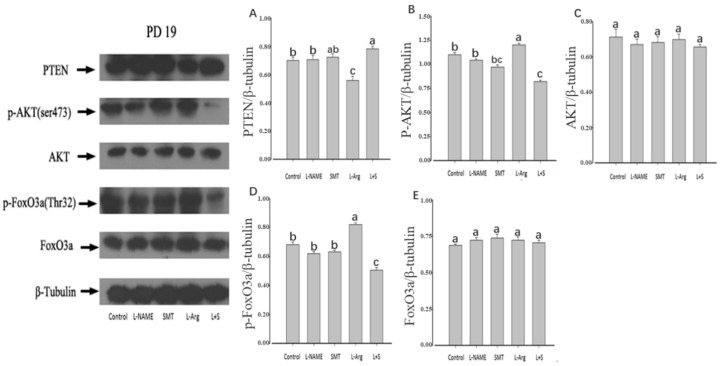
Effect of NOS inhibitors and substrate on ovarian expression of PTEN (**A**), p-AKT (**B**), AKT (**C**), p-FoxO3a (**D**), and FoxO3a (**E**) in immature rats. Animals in the control, L-NAME, SMT, L-Arg, or L + S (L-NAME plus SMT) groups were subcutaneously hypodermically injected with PBS, L-NG-Nitroarginine Methyl Ester (40 mg/kg), S-Methylisothiourea (10 mg/kg), L-NG-Nitroarginine Methyl Ester (40 mg/kg) and S-Methylisothiourea (10 mg/kg), or L-arginine (50 mg/kg) for 19 days (PD19) from the first day of birth (PD1). As an internal control, β-tubulin was used, wherein the signal intensity was plotted as the ratio of target protein to β-tubulin. Bars with different letters denote significant differences (*p* < 0.05).

The injection of L-NAME or SMT resulted no significant difference of the ovarian expression of PTEN, p-AKT, AKT, p-FoxO3a, and FoxO3a on PD5, PD10, and PD19 in comparison with the control (*p* > 0.05, Figure 3, Figure 4 and Figure 5). Furthermore, no significant differences were observed in the ovarian expressions of AKT and FoxO3a on PD5, PD10, and PD10 among all groups (*p* > 0.05, Figure 3C,E, Figure 4C,E and Figure 5C,E).

Compared with the control group, the injection of L-Arg resulted in no significant difference in the ovarian expression of PTEN, p-AKT, and p-FoxO3a on PD5 (*p* > 0.05, Figure 3A,B,D). The ovarian expression of p-AKT and p-FoxO3a in the L-Arg group was significantly upregulated at PD10 (*p* < 0.05, Figure 4B,D), and the ovarian expression of PTEN in the L-Arg group was significantly downregulated on PD19, with significant upregulation of the ovarian expression of p-AKT and p-FoxO3a (*p* < 0.05, Figure 5A,B,D). Additionally, the rats in the L-Arg group showed significantly lower expression of PTEN and significantly higher expression of p-AKT and p-FoxO3a on PD5, PD10, and PD19 in comparison with animals injected with L-NAME plus SMT (*p* < 0.05, Figure 3A,B,D, Figure 4A,B,D and Figure 5A,B,D).

Compared with the control group, the ovarian expression of p-AKT in the L + S group was significantly downregulated on PD5 (*p* < 0.05, Figure 3B), and the ovarian expression of PTEN in the L + S group was significantly upregulated on PD10, with the significant downregulation of the ovarian expression of p-FoxO3a (*p* < 0.05, Figure 4A,D). The ovarian expression of PTEN in the L + S group was significantly upregulated on PD19, with significant downregulation of the ovarian expression of p-AKT and p-FoxO3a (*p* < 0.05, Figure 5A,B,D).

### 3.4. Ovarian Expression of LC3-II and Cleaved-Caspase-3

**Figure 6:**
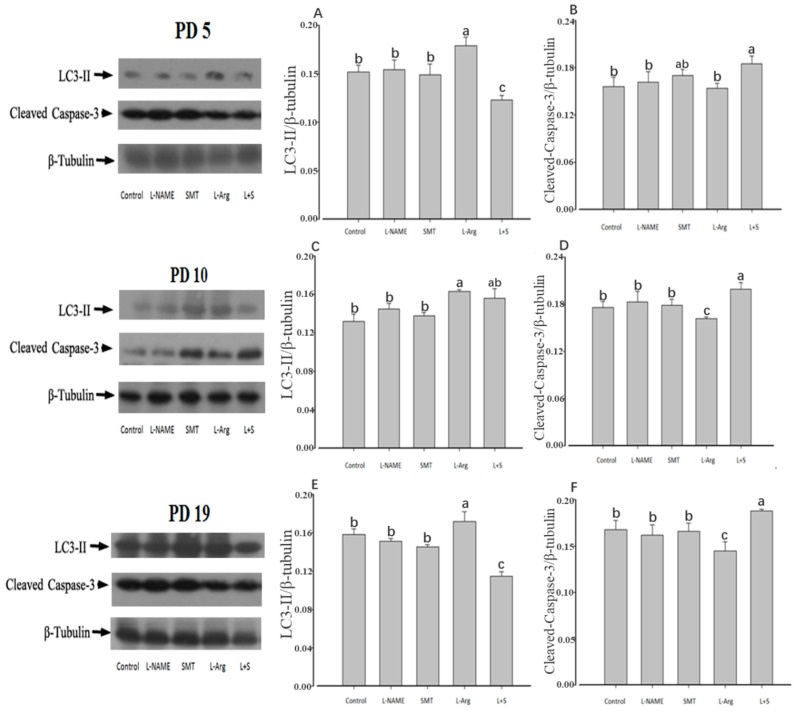
Effect of NOS inhibitors and substrate on ovarian expression of LC3-II (**A**,**C**,**E**) and cleaved-Caspase-3 (**B**,**D**,**F**) in neonatal and immature rats. Animals in the control, L-NAME, SMT, L-Arg, or L + S (L-NAME plus SMT) groups were subcutaneously hypodermically injected with PBS, L-NG-Nitroarginine Methyl Ester (40 mg/kg), S-Methylisothiourea (10 mg/kg), L-NG-Nitroarginine Methyl Ester (40 mg/kg) and S-Methylisothiourea (10 mg/kg), or L-arginine (50 mg/kg) for five (PD5), 10 (PD10) and 19 days (PD19) from the first day of birth (PD1). As an internal control, β-tubulin was used, wherein the signal intensity was plotted as the ratio of target protein to β-tubulin. Bars with different letters denote significant differences (*p* < 0.05).

This study showed that there were no significant differences in the expression patterns of LC3-II and cleaved Caspase-3 among the control, L-NAME, and SMT groups on PD5, PD10, or PD19 (*p* > 0.05, Figure 6A–F). Compared with the control group, the ovarian expression of LC3-II in the L-Arg group was significantly upregulated on PD5, PD10, and PD19 (*p* < 0.05, Figure 6A,C,E), while the ovarian expression of cleaved Caspase-3 was significantly downregulated on PD10 and PD19 (*p* < 0.05, Figure 6D,F). In contrast, the ovarian expression of LC3-II in the L + S group was significantly downregulated on PD5 and PD19 (*p* < 0.05, Figure 6A,E), while the ovarian expression of cleaved Caspase-3 was significantly upregulated on PD5, PD10, and PD19 (*p* < 0.05, Figure 6B,D,F).

## 4. Discussion

The complexity of ovarian internal factors regulating follicular development and oocyte maturity requires further investigation [48]. To address this, NOS inhibition models were established via injection of L-NAME (40 mg/kg) and SMT (10 mg/kg) in vivo as selective inhibitors of eNOS and iNOS, respectively. The results revealed that follicular morphology and NOS activity were not significantly impaired by a single NOS inhibitor injection of L-NAME or SMT; the injection concentration of L-NAME or SMT may have been too low to affect the activity of selective eNOS or iNOS [10], and a higher level of inhibitor could induce death. Therefore, L-NAME plus SMT might contribute to synergistic inhibition, and we found that it significantly decreased the NOS activity and NO content in neonatal and immature rats. Furthermore, L-Arg promoted NO generation and induced advanced follicular development. This is consistent with the finding that the ratio between NO synthesis boosters and inhibitors is higher in ewes carrying multiple fetuses [49].

A previous study reported that sGC, a cGMP synthesizing enzyme, is mainly expressed in the granulosa cells of primordial follicles and preantral follicles, indicating possible associations between the cGMP signal transduction pathway with the initiation of follicle growth [50]. The fact that FoxO3 is over-phosphorylated with PI3K and ATK activation induced by PTEN-deficient oocytes indicates the possibility that the PI3K/FoxO3 pathway is the key factor regulating the activation of primordial follicles [25,27]. During the migration of endothelial cells, NO downregulates peroxisome proliferator by activating the PI3K/AKT pathway and inactivating FoxO3a protein, thereby inhibiting and downregulating PGC-1α [31]. The above mechanisms may explain our observation that the percentage of antral follicle is significantly elevated or decreased after injection of L-Arg or L + S, respectively. Consistently, NOS inhibition resulted in the suppression of NO synthesis and antral follicle development [8]. However, the NOS substrate and inhibitor has different influence across ages, and injection of L-Arg significantly increased or decreased the secondary follicle (SF) percentage on PD10 or PD19 respectively, since the NOS substrate or inhibitor might contribute to different functions of the ovary at different stages. For example, NO is produced by iNOS only in immature follicles at earlier stages in rats [18].

Furthermore, previous studies have found that the NO/cGMP pathway participated in the follicle development of rats and porcine animals [34,35]. Therefore, the current investigation was designed to reveal the relationship between the NO/cGMP and PI3K pathways during follicular development in neonatal rats, particularly in the immature period from birth to puberty. The activity of FoxOs is tightly regulated by posttranslational modification, including phosphorylation, acetylation, and ubiquitylation [51]. The p-FoxO3a proteins are positioned in the oocyte nucleolus of primordial and primary follicles, and transfer to the cytoplasts in secondary and mature follicles, thus possibly initiating follicular development [52]. Our study showed that the key proteins of the PI3K/AKT/FoxO3a pathway, including PTEN, AKT, and FoxO3a, were highly expressed in the ovary on PD5, PD10, and PD19. This finding is consistent with the expression of key proteins in oocyte and granulosa cells in adult animals, where p-AKT is expressed in adult porcine ovarian granulosa cells of medium follicles associated with autophagy [53], and follicle loss has been ascribed to the activation of the PTEN/PI3K/AKT pathway in adult female mice [54].

As reported, NOS/cGMP activated the PI3K/AKT pathway [55], which is involved in chemotherapy-induced follicular activation and responds to fragmentation [56]. In addition, the NOS/cGMP pathway is related to the angiotensin (HIF-1) in endothelial cells [57]. Therefore, NOS/cGMP is engaged via numerous routes in the PI3K/AKT pathway, through it remains unclear whether it can further activate FoxO3a, especially during ovary development in female neonatal rats. Compared with the control group on PD19, Western blotting analysis showed that the NOS substrate induced downregulation of PTEN and upregulation of p-AKT and p-FoxO3a, while the combined NOS inhibitors upregulated PTEN and downregulated p-AKT and p-FoxO3a. There is the possibility that NOS might activate the PI3K/AKT/FoxO3a pathway via downregulation of PTEN and upregulation of p-AKT and p-FoxO3a during follicular development in immature rats. However, how NOS mediates the PI3K/AKT/FoxO3a pathway through cGMP and regulates follicular development requires further investigation.

This study showed that the apoptosis-marker protein of cleaved Caspase3 and the autophagia-marker protein of LC3-II were highly expressed in the ovary on PD5, PD10, and PD19, which was consistent with previous studies [58]. The participation of apoptosis and autophagia in follicular development was contributed to by oocyte atresia [59,60]. Furthermore, NO has been proven to function in cell autophagia and apoptosis [61,62,63], wherein high-content NO could accelerate oocyte atresia by producing excessive superoxide or inducing apoptosis [64,65]. The current study confirmed the role of NOS in the ovarian development of neonatal and immature female rats via cell autophagia and apoptosis, with upregulation of LC3-II and downregulation of cleaved Caspase-3 of L-Arg injected rats on PD19 along with elevated activity of NOS and content of NO in the ovary, while the injection of L-NAME plus SMT showed the opposite regulation of LC3-II and cleaved Caspase-3 in the ovary.

## 5. Conclusions

In summary, daily exposure to NOS subcutaneous administration in female neonatal and immature rats proficiently contributed to NO generation and follicular development for 19 consecutive days. The NOS substrate contribution induced downregulation of PTEN and upregulation of p-AKT and p-FoxO3a, which are associated with upregulation of LC3-II and downregulation of cleaved Caspase-3 during follicular autophagy and apoptosis in neonatal and immature female rats respectively. This study provides a novel perspective in understanding progressive follicular development using NOS substrates. These insights may serve as a reference for further investigation of the mechanism involved in the progressive follicular development of antral follicles in the ovaries.
